# 2,3,4′,5-Tetrahydroxystilbene-2-*O*-β-D-Glucoside (THSG) Activates the Nrf2 Antioxidant Pathway and Attenuates Oxidative Stress-Induced Cell Death in Mouse Cochlear UB/OC-2 Cells

**DOI:** 10.3390/biom10030465

**Published:** 2020-03-18

**Authors:** Tien-Yuan Wu, Jia-Ni Lin, Zi-Yao Luo, Chuan-Jen Hsu, Jen-Shu Wang, Hung-Pin Wu

**Affiliations:** 1 Department of Pharmacology, School of Medicine, College of Medicine, Tzu Chi University, Hualien 970, Taiwan; tyuanwu@gms.tcu.edu.tw; 2 Department of Pharmacy, Taichung Tzu Chi Hospital, Buddhist Tzu Chi Medical Foundation, Taichung 427, Taiwan; 3 Department of Otolaryngology Head and Neck Surgery, Taichung Tzu Chi Hospital, Buddhist Tzu Chi Medical Foundation, Taichung 427, Taiwan; neonlin0939@gmail.com (J.-N.L.); harris207tw@gmail.com (Z.-Y.L.); cjhsu@ntu.edu.tw (C.-J.H.); 4 School of Medicine, Tzu Chi University, Hualien 970, Taiwan; wang0826@tzuchi.com.tw; 5 Department of Otolaryngology, National Taiwan University Hospital, Taipei 100, Taiwan; 6 Department of Chinese Medicine, Taichung Tzu Chi Hospital, Buddhist Tzu Chi Medical Foundation, Taichung 427, Taiwan

**Keywords:** 2,3,4′,5-tetrahydroxystilbene-2-*O*-β-D-glucoside (THSG), mouse cochlear UB/OC-2 cells, apoptosis, autophagy, nucleus factor erythroid 2-related factor 2 (Nrf2)

## Abstract

Oxidative stress plays a critical role in the pathogenesis of hearing loss, and 2,3,4′,5-tetrahydroxystilbene-2-*O*-β-D-glucoside (THSG) exerts antioxidant effects by inhibiting reactive oxygen species (ROS) generation. With the aim of developing new therapeutic strategies for oxidative stress, this study investigated the protective mechanism of THSG in vitro using a normal mouse cochlear cell line (UB/OC-2). The THSG and ascorbic acid have similar free radical scavenger capacities. H_2_O_2_, but not THSG, reduced the UB/OC-2 cell viability. Moreover, H_2_O_2_ might induce apoptosis and autophagy by inducing morphological changes, as visualized by microscopy. As evidenced by Western blot analysis and monodansylcadaverine (MDC) staining, THSG might decrease H_2_O_2_-induced autophagy. According to a Western blotting analysis and Annexin V/PI and JC-1 staining, THSG might protect cells from H_2_O_2_-induced apoptosis and stabilize the mitochondrial membrane potential. Furthermore, THSG enhanced the translocation of nucleus factor erythroid 2-related factor 2 (Nrf2) into the nucleus and increased the mRNA and protein expression of antioxidant/detoxifying enzymes under H_2_O_2_-induced oxidative stress conditions. Collectively, our findings demonstrate that THSG, as a scavenging agent, can directly attenuate free radicals and upregulate antioxidant/detoxifying enzymes to protect against oxidative damage and show that THSG protects UB/OC-2 cells from H_2_O_2_-induced autophagy and apoptosis in vitro.

## 1. Introduction

Hearing loss is a serious global morbidity that affects 360 million people worldwide [1], and this neurological disability impacts both the physical and mental health of patients [2]. Recent research has revealed several mechanisms and molecules that contribute to hearing loss, such as oxidative stress and apoptotic cell death [3]. Oxidative stress plays a critical role in the pathogenesis of hearing loss and in diabetes-related and environmental/occupational exposure-associated complications [4,5].

The available evidence suggests that excessive oxidative stress in the cochlea is closely related to the pathogenesis of hearing loss [3,6]. Therefore, pharmacologically reducing oxidative stress and strengthening the cochlear resistance against oxidative stress and cytotoxic chemicals are effective prevention strategies for maintaining the structural and functional integrity of the cochlea to prevent the development and progression of hearing loss.

An increasing body of evidence shows that the consumption of dietary phytochemicals and herbal supplements as antioxidant agents can reduce intracellular oxidative stress and free radicals [7,8,9,10,11,12]. Cellular and molecular studies have revealed that chemopreventive phytochemicals appear to exert cytoprotective effects on normal cells through the activation of the nucleus factor erythroid 2-related factor 2 (Nrf2) signaling pathway [7,8,9,12,13,14]. The Nrf2 signaling pathway plays a vital role in cells against oxidative stress. Under stress conditions, Nrf2 translocates from the cytoplasm to the nucleus and then binds to the antioxidant response element (ARE), resulting in the activation of several antioxidant genes, such as heme oxygenase-1 (HO-1), NAD(P)H:quinone oxidoreductase-1 (NQO-1), UDP-glucuronosyltransferase (UGT), and glutathione-S-transferase (GST) [15].

Mitochondria are a major source of endogenous reactive oxygen species (ROS), and a progressive decline in mitochondrial function results in the increased production of ROS, which leads to oxidative damage and dysfunction in various tissues [16,17]. The depolarization of the mitochondrial membrane potential following excessive ROS generation leads to activation of the mitochondria-dependent apoptosis pathway. Caspase 9, the initiator caspase in the mitochondria-dependent apoptosis pathway, activates the downstream effector Caspase 3, and active Caspase 3 impairs DNA repair through the cleavage of poly ADP-ribose polymerase (PARP). Excess ROS upregulate autophagy to remove proteins and organelles with oxidative damage in the cytoplasm [18,19,20,21]. LC3-phosphatidylethanolamine conjugate (LC3-II), a marker for autophagy, is involved in the formation of autophagosome membranes, and autophagosomes might fuse with lysosomes to degrade damaged cytosolic components [22,23,24,25].

2,3,4′,5-Tetrahydroxystilbene-2-*O*-β-D-glucoside (THSG), a major component of *Polygonum multiflorum* Thunb. (He-Shou-Wu), has been shown to have various health benefits. The neuroprotective effect of THSG on glutamate-induced hippocampal damage decreases ROS production and stabilizes the mitochondrial membrane potential [14]. Upregulation of the Nrf2 signaling pathway and downregulation of the nuclear factor-κB (NF-κB) signaling pathway can attenuate oxidative injury in osteoblasts [26]. The THSG exhibits antidepressant efficacy by modulating oxidative stress and inflammatory pathways [27]. We conducted this study to investigate the protective mechanism of THSG in vitro using a normal mouse cochlear cell line (UB/OC-2), and the results will hopefully aid the development of new therapeutic strategies for oxidative stress. 

## 2. Materials and Methods

### 2.1. Chemical Reagents and Antibodies

Hydrogen peroxide (H_2_O_2_), ascorbic acid, and 1,1-diphenyl-2-picrylhydrazyl radical 2,2-diphenyl-1-(2,4,6-trinitrophenyl)hydrazyl (DPPH) were purchased from Sigma*-*Aldrich (St. Louis, MO, USA). 3-(4,5-Dimethylthiazol-2-yl)-2,5-diphenyltetrazolium bromide (MTT) was purchased from VWR International (Radnor, PA, USA). Primary antibodies against cleaved PARP, caspase 9, cleaved caspase 3, LC3, β-actin, lamin B, and HRP-linked secondary antibodies were purchased from Cell Signaling Technology (Beverly, MA, USA). Primary antibodies against Nrf2, HO-1, UGT, and GST and the cell fractionation kit were purchased from Abcam (Cambridge, MA, USA). The THSG was purchased from MedChemExpress Company (Monmouth Junction, NJ, USA), and 5,5,6,6′-tetrachloro-1,1′,3,3′-tetraethylbenzimidazolylcarbocyanine iodide (JC-1) and Annexin V/PI apoptosis kits were purchased from Enzo Life Sciences (Farmingdale, NY, USA). TRIzol^®^ and SuperScript^®^ III Reverse Transcriptase were purchased from Invitrogen (Carlsbad, CA, USA), and the QuantiNova^®^ SYBR Green PCR Kit was obtained from Qiagen (Valencia, CA, USA). 

### 2.2. Determination of Antioxidant Activity with the 1,1-Diphenyl-2-Picrylhydrazyl Radical 2,2-Diphenyl-1-(2,4,6-Trinitrophenyl)hydrazyl (DPPH) Radical Scavenging Assay

In 96-well plates, 20 μL of each sample (6.25–200 μM) was mixed with 80 μL of 100 mM Tris-HCl buffer at pH 7.4 and 100 μL of 200 μM DPPH ethanol solution. The mixtures were incubated at room temperature (RT) for 20 min in darkness, and the absorbance at 517 nm was then measured using a microplate reader (Infinite 200 PRO Series Multimode Reader; TECAN, Switzerland). The DPPH radical scavenging effect was calculated using the following equation: [(A0–As)/A0] × 100, where A0 is the absorbance of the control reaction and As is the absorbance of the sample. The experiments were performed in triplicate.

### 2.3. Cell Culture and Cell Viability Assay

The mouse cochlear UB/OC-2 cell line was obtained from Ximbio (London, UK). The cells were cultured in minimal essential medium with Earle’s salts and GlutaMAX (Gibco; Thermo Fisher Scientific, Inc., Waltham, MA, USA) supplemented with 10% FBS (HyClone Laboratories Inc.; Logan, UT, USA) and 50 U/mL IFN-γ (R&D Systems; Minneapolis, MN, USA) at 33 °C under a humidified atmosphere consisting of 95% air and 5% CO_2_.

The changes in cellular morphology were observed using an Olympus BX41 microscope (Tokyo, Japan), and the MTT assay was used for the assessment of cell viability. Three hours before the end of the incubation period, 40 μL of MTT solution (2 mg/mL) was added to each well. The formazan product that was formed during the reaction was dissolved in DMSO. The results were measured using a microplate reader at 590 nm. The experiments were performed in triplicate.

### 2.4. Cell Fractionation

Cell fractionation was performed according to the manufacturer’s instructions. The cells were collected by centrifugation at 1200 rpm for 3 min and then washed once with ice-cold PBS. The collected cells were counted and resuspended in Buffer A (containing 0.36% Tris and 0.015% ethylenediaminetetraacetic acid (EDTA)) to 2 × 10^7^ cells/mL. An equal volume of Buffer B (containing 0.001% digitonin, 0.36% Tis, and 0.015% EDTA) was added, and the mixture was incubated for 7 min on a rotator at RT. The samples were centrifuged at 5000 rpm for 1 min, and the cytosolic fractions in the resulting supernatants were collected into new tubes. The pellets were resuspended in the same volume of Buffer A. An equal volume of Buffer C (containing 0.3744% Tris and 0.0157% EDTA) was then added, and the resulting mixture was incubated for 10 min on a rotator at RT. The samples were centrifuged at 10,000 rpm for 1 min, and the supernatants were removed (mitochondrial fractions). The pellets containing the nuclear fractions were resuspended in Buffer A.

### 2.5. Western Blotting Analysis

The cells were suspended in protein extraction solution (iNtRON Biotechnology; Burlington, MA, USA). After incubation on ice for 30 min, the cell lysates were cleared by centrifugation at 12,000 rpm and 4 °C for 20 min. The lysate (30 μg) was subjected to 8–15% SDS-polyacrylamide gel electrophoresis, and the proteins were transferred to polyvinylidene difluoride membranes using a wet transfer system. The membranes were incubated with 3% BSA in Tris-buffered saline (TBS) for 1 h at RT and then probed with the primary antibody overnight at 4 °C with gentle shaking. The next day, the membranes were incubated with horseradish peroxidase-conjugated secondary antibody for 1 h. The images of the membranes were developed by enhanced chemiluminescence (EMD Millipore; Schwalbach, Germany) using a KETA C Chemi imaging system (Wealtec Corporation; Sparks, NV, USA).

### 2.6. Monodansylcadaverine (MDC) Staining

After treatment, the cells were stained with 0.05 mM MDC for 15 min at 33 °C, washed twice with phosphate-buffered saline (PBS), and immediately observed under a microscope. The autophagic vacuoles were detected at 335 nm using a 335-nm excitation filter and a 420-nm emission filter, and the resulting fluorescent images were observed using an Olympus BX41 microscope.

### 2.7. Transmission Electron Microscopy (TEM)

The cells were fixed with 2.5% glutaraldehyde in 0.1 M cacodylate buffer overnight at 4 °C and postfixed in 1% osmium tetroxide in 0.1 M cacodylate buffer for 1 h. The cells were then stained with 2% uranyl acetate and subjected to gradient dehydration with ethanol-acetone. The cells were then embedded in Spurr’s resin for sectioning. Images were obtained using a Hitachi H-7500 transmission electron microscope (Tokyo, Japan).

### 2.8. Annexin V/PI Staining

To assess the effect on cell death, the apoptotic cells were quantified using an Annexin V/PI apoptosis kit. The cells were washed twice with ice-cold PBS and resuspended in 400 μL of binding buffer, and 2.5 μL of Annexin V and 5 μL of PI were then added. The mixture was mixed properly and incubated for 15 min at 4 °C in the dark. A BD Accuri™ C6 flow cytometry system (BD Biosciences; San Jose, CA, USA) was used for the analysis of cell death.

### 2.9. 5,5,6,6’-Tetrachloro-1,1’,3,3’-Tetraethylbenzimidazolylcarbocyanine Iodide (JC-1) Staining

The JC-1 staining was used to assess the changes in the mitochondrial membrane potential. The cells were incubated with 5 μg/mL JC-1 solution for 10 min at 33 ℃ and washed twice with PBS. The fluorescence was detected using a 515-nm excitation filter and a 529-nm emission filter, and the images were obtained with an Olympus BX41 fluorescence microscope.

### 2.10. Real-Time Polymerase Chain Reaction

The cells were washed twice with ice-cold PBS, and TRIzol reagent was then added for the isolation of total RNA. Total RNA was converted to cDNA using SuperScript III Reverse Transcriptase according to the manufacturer’s recommended protocol. Gene expression was measured using the SYBR Green dye (an asymmetrical cyanine dye) method and analyzed with a StepOnePlus Real-Time PCR System (Applied Biosystems; Foster City, CA, USA).

The following primers were used: Nrf2 (sense 5’- CATGATGGACTTGGAGTTGC-3´ and antisense 5´-CCTCCAAAGGATGTCAATCAA-3´), NQO-1 (sense 5´-AGCGTTCGGTATTACGATCC-3´ and antisense 5´-AGTACAATCAGGGCTCTTCTCG-3´), HO-1 (sense 5´-GGGTGATAGAAGAGGCCAAGA-3´ and antisense 5´-AGCTCCTGCAACTCCTCAAA-3´) and GAPDH (sense 5´-GCCAAAAGGGTCATCATCTC-3´ and antisense 5´-CACACCCATCACAAACATGG-3´).

### 2.11. Statistics

The data are expressed as the means ± standard deviations (SDs). Student’s *t* test was used to compare the means between two groups. A *p* value less than 0.05 was considered statistically significant.

## 3. Results

3.1. 2,3,4′,5-Tetrahydroxystilbene-2-O-β-D-Glucoside Has Free Radical Scavenger Capacity

**Figure 1:**
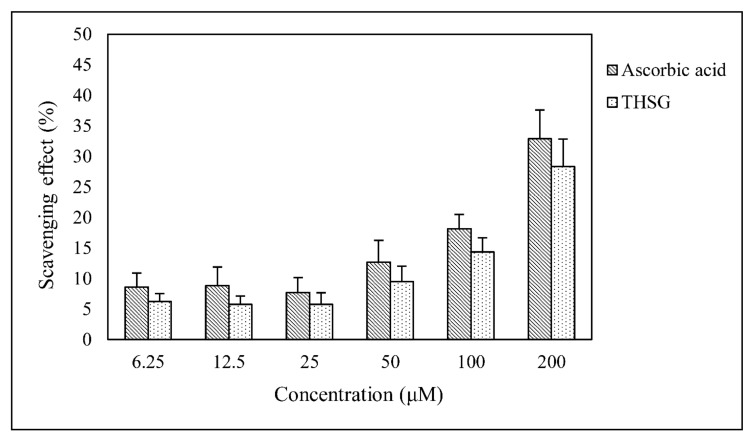
Effect of 2,3,4′,5-Tetrahydroxystilbene-2-*O*-β-D-glucoside (THSG) (6.25–200 μM) on 2,2-diphenyl-1-(2,4,6-trinitrophenyl)hydrazyl (DPPH) free radical scavenging activity. Ascorbic acid was used as a positive control. Data are presented as the mean ± SD, *n* = 3.

The DPPH free radical scavenging assay was used to examine the free radical scavenger activity of THSG, and ascorbic acid was used as a positive control (Figure 1). Over a concentration gradient of 10% to 30%, the ability of THSG to scavenge free radicals was similar to that of ascorbic acid. No significant difference was observed between THSG and ascorbic acid, which suggests that the ability of THSG to scavenge free radicals was comparable to that of ascorbic acid.

### 3.2. H_2_O_2_ But Not 2,3,4′,5-Tetrahydroxystilbene-2-O-β-D-Glucoside Reduces Cell Viability

**Figure 2:**
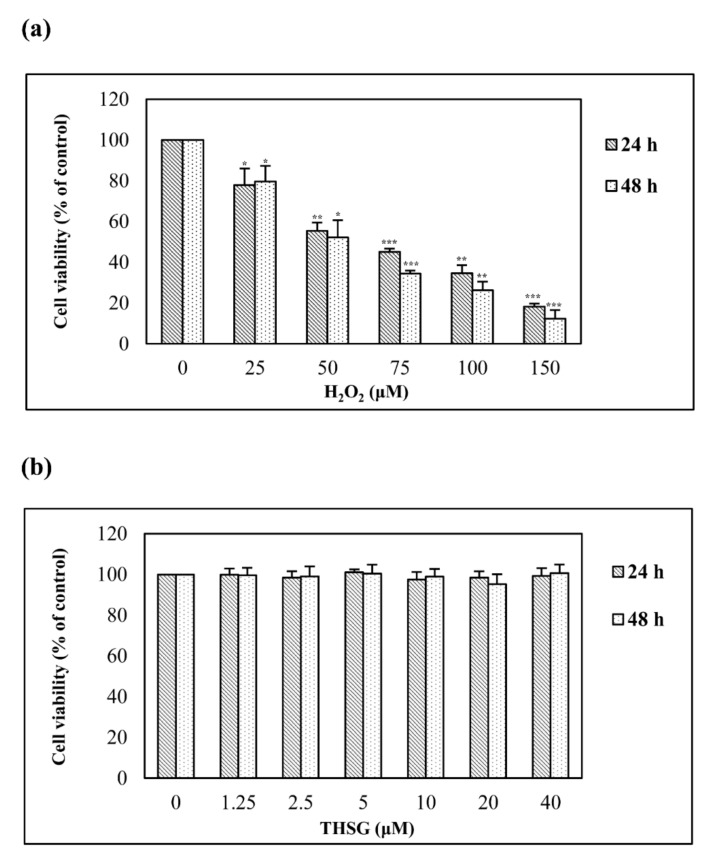
H_2_O_2_ but not THSG reduces cell viability. The cells were treated with various concentrations of H_2_O_2_ (0–150 μM) (**a**) or THSG (0–40 μM) (**b**) for 24 and 48 h, and their viability was determined using a 3-(4,5-dimethylthiazol-2-yl)-2,5-diphenyltetrazolium bromide (MTT) assay. The cell viability of untreated cells was set to 100%. Data are presented as the mean ± SD, *n* = 3. *p < 0.05, **p < 0.01, and ***p < 0.001 indicate significant differences compared with the untreated group.

H_2_O_2_ is the oxidizing agent that has most commonly been used to investigate the response of cells to oxidative stress. UB/OC-2 cells were stimulated with various concentrations (0, 25, 50, 75, 100, or 150 μM) of H_2_O_2_ for 24 and 48 h, and the resulting cell viability was measured using the MTT assay. The H_2_O_2_ treatment induced a time- and concentration-dependent reduction in UB/OC-2 cell viability (Figure 2a). Only a low H_2_O_2_ concentration (25 μM) resulted in a higher cell viability at 48 h than at 24 h, but this increase was not significant, and this result might be due to sampling error. The IC_50_ of H_2_O_2_-induced UB/OC-2 cells obtained with both the 24- and 48-h treatments was higher than 50 μM. Therefore, 75 μM H_2_O_2_ was used in this study. An MTT assay was used to examine the cytotoxicity of THSG. The survival of UB/OC-2 cells treated with THSG at different concentrations (0–40 μM) for 24 and 48 h was almost 100% (Figure 2b).

### 3.3. H_2_O_2_ Might Induce Apoptosis and Autophagy

**Figure 3:**
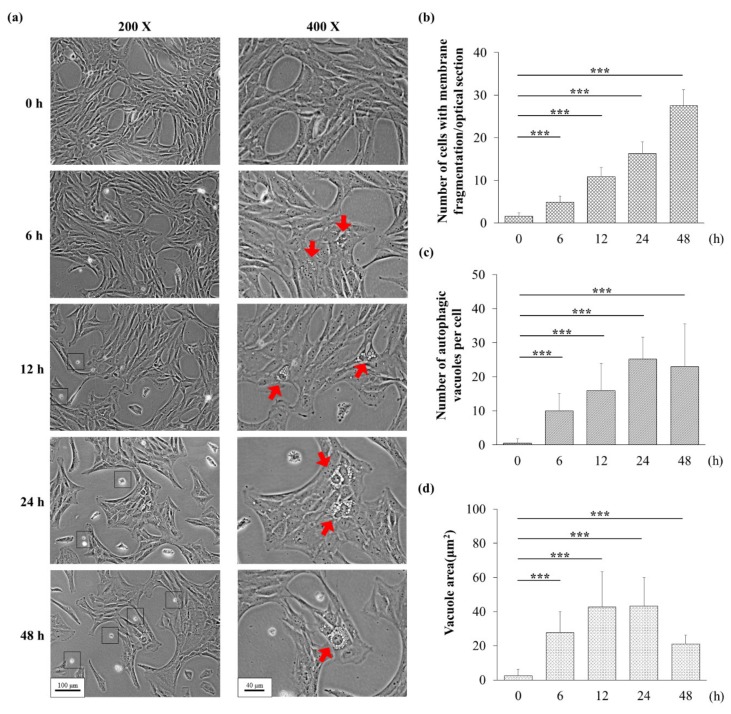
Effects of H_2_O_2_ in mouse cochlear UB/OC-2 cells. (**a**) The cells were treated with 75 μM H_2_O_2_ for 24 and 48 h, and images were obtained at the end of the incubation period using a visible light microscope (black bar: magnification of 200 X =100 μm and magnification of 400 X = 40 μm). Squares indicate membrane fragmentation and red arrows indicate cytoplasmic vacuoles. (**b**) The number of cells with membrane fragmentation was counted in six randomly optical sections. (**c**) The number of autophagic vacuoles per cell was analyzed from at least 10 randomly chosen fields. (**d**) The area of autophagic vacuole was analyzed from at least 10 randomly chosen cells. For panels (**b**–**d**), *p < 0.05, **p < 0.01, and *** *p* < 0.001 indicate significant differences from the control group.

Treatment with 75 μM H_2_O_2_ induced morphological changes in UB/OC-2 cells (Figure 3a). Many vacuoles were found in the cytoplasm after 6 h of exposure to H_2_O_2_, and these findings prompted us to examine whether autophagy was activated after exposure to H_2_O_2_. In addition, membrane fragmentation was observed in UB/OC-2 cells during exposure to H_2_O_2_. Cells with membrane fragmentation and autophagic vacuoles are shown with arrows in Figure 3a. We thus speculated that H_2_O_2_ might induce apoptosis and autophagy.

### 3.4. 2,3,4′,5-Tetrahydroxystilbene-2-O-β-D-Glucoside Inhibits H_2_O_2_-Induced Autophagy

**Figure 4:**
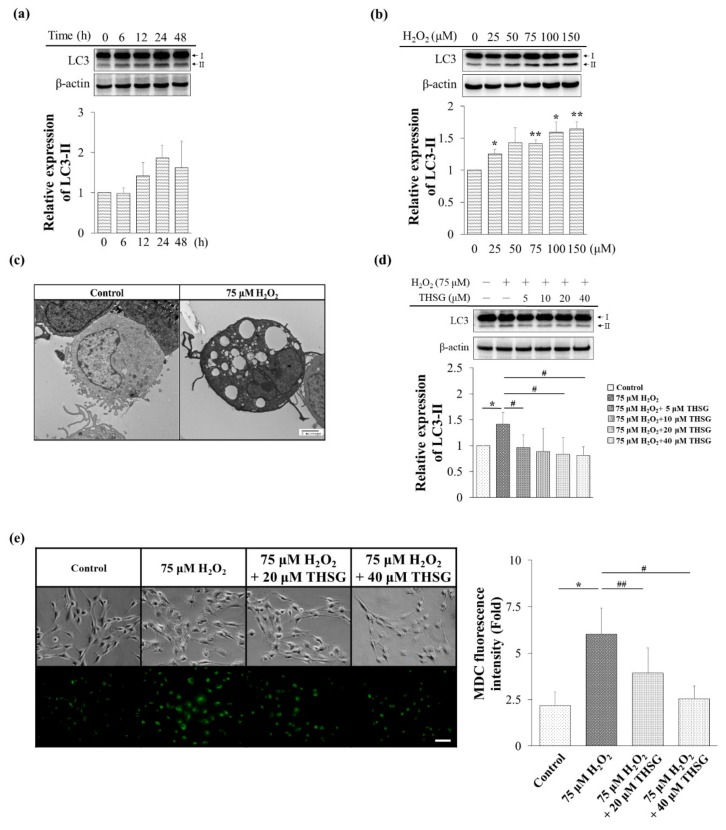
The THSG inhibits H_2_O_2_-induced autophagy in mouse cochlear UB/OC-2 cells. (**a**) The cells were treated with 75 μM H_2_O_2_ for 0–48 h, and the protein level of LC3 was measured by Western blotting. (**b**) The cells were treated with various concentrations of H_2_O_2_ (0–150 μM) for 24 h, and the protein level of LC3 was measured by Western blotting. (**c**) The cells were treated with 75 μM H_2_O_2_ for 18 h, and autophagy was observed by TEM. Black bar = 2 μm. (**d**) The cells were pretreated with various concentrations of THSG (5–40 μM) for 6 h and then stimulated with 75 μM H_2_O_2_ for 24 h, and the protein level of LC3 was measured by Western blotting. β-actin was used as the loading control. The results of protein expression were normalized to β-actin. (**e**) The cells were pretreated with 20 or 40 μM THSG for 6 h and subsequently with 75 μM H_2_O_2_ for 24 h, and the fluorescence obtained using the MDC dye was detected using a fluorescence microscope. Scale bar = 40 μm. Data are presented as the mean ± SD, *n* = 3. *p < 0.05 and **p < 0.01 indicate significant differences from the control group. **^#^***p* < 0.05 and **^##^***p* < 0.01 indicate significant differences from the H_2_O_2_-treated group.

The LC3-II is an autophagic marker that is recruited to the autophagosome membrane. The LC3-II protein level was estimated after treatment with 75 μM H_2_O_2_ for 0–48 h in UB/OC-2 cells. As shown in Figure 4a, the LC3-II protein level at a concentration of 75 μM H_2_O_2_ significantly increased after 24 h of treatment. H_2_O_2_ (0–150 μM) increased the LC3-II protein level in a concentration-dependent manner (Figure 4b).

Transmission electron microscopy (TEM) revealed that the treatment of UB/OC-2 cells with 75 µM H_2_O_2_ for 18 h might result in vacuole formation (Figure 4c). The LC3-II protein level was decreased in the THSG (5–40 μM)-treated groups (Figure 4d). In addition, the number of MDC-labeled vacuoles was decreased in the 20 and 40 μM THSG-treated groups compared with the H_2_O_2_-treated group (Figure 4e). Taken together, these results show that THSG protects against the H_2_O_2_-induced autophagy in UB/OC-2 cells.

### 3.5. 2,3,4′,5-Tetrahydroxystilbene-2-O-β-D-Glucoside Protects against H_2_O_2_-Induced Apoptosis

**Figure 5:**
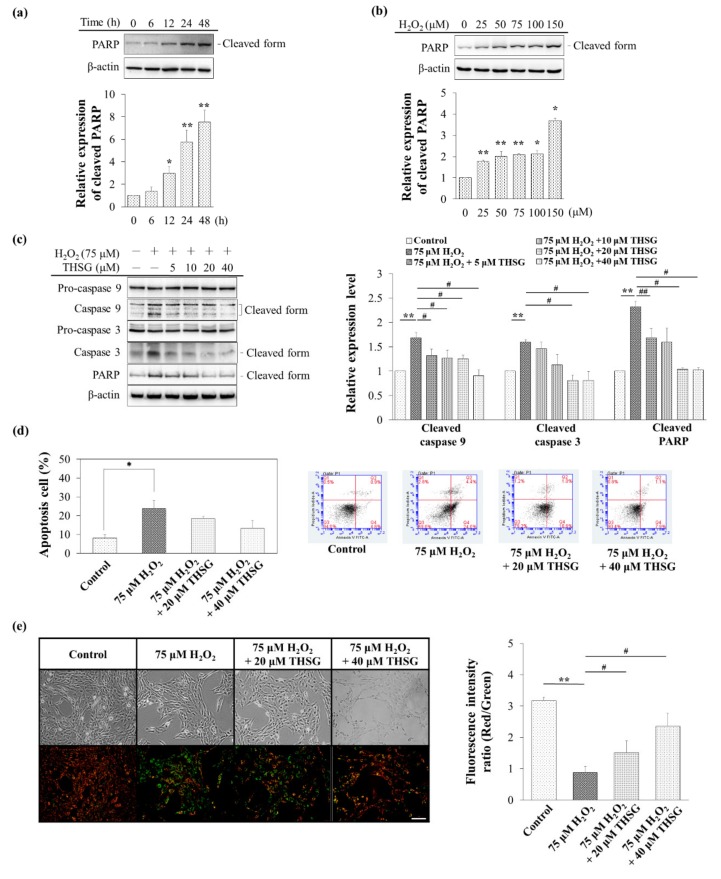
The THSG protects against H_2_O_2_-induced apoptosis. (**a**) The cells were treated with 75 μM H_2_O_2_ for 0–48 h, and the protein level of cleaved PARP was determined by Western blotting. (**b**) The cells were treated with various concentrations of H_2_O_2_ (0–150 μM) for 48 h, and the protein level of cleaved PARP was measured by Western blotting. (**c**) The cells were pretreated with various concentrations of THSG (5–40 μM) for 6 h and subsequently with 75 μM H_2_O_2_ for 48 h, and the protein levels of pro-caspase 9, cleaved caspase 9, pro-caspase 3, cleaved caspase 3, and cleaved PARP were measured by Western blotting. β-actin was used as the loading control. The results of protein expression were normalized to β-actin. (**d**) The cells were pretreated with 20 or 40 μM THSG for 6 h and subsequently with 75 μM H_2_O_2_ for 24 h, and the apoptotic cells were subjected to Annexin V/PI staining and analyzed by flow cytometry. (**e**) The cells were pretreated with 20 or 40 μM THSG for 6 h and subsequently with 75 μM H_2_O_2_ for 24 h, and the fluorescence obtained with the JC-1 dye was detected by fluorescence microscopy. Scale bar = 100 μm. Data are presented as the mean ± SD, *n* = 3. *p < 0.05 and **p < 0.01 indicate significant differences from the control group. **^#^***p* < 0.05 and **^##^***p* < 0.01 indicate significant differences from the H_2_O_2_-treated group.

H_2_O_2_ increased the production of ROS, which diffused into the cytosol to activate the mitochondria-dependent apoptosis pathway and thus activated cleaved PARP, and these effects were both time (6, 12, 24, and 48 h; Figure 5a)- and concentration (0–150 μM H_2_O_2_; Figure 5b)-dependent. THSG (5–40 μM) protected against H_2_O_2_ damage and reduced the cleaved caspase 9, cleaved caspase 3, and cleaved PARP levels (Figure 5c). A flow cytometry analysis with Annexin V/PI staining revealed that THSG protected UB/OC-2 cells against H_2_O_2_ (75 μM for 24 h)-induced apoptosis. The percentage of apoptotic cells was calculated as the sum of early and late apoptotic cells. In addition, 20 and 40 μM THSG significantly attenuated the percentage of apoptotic cells compared with those obtained with H_2_O_2_ treatment (Figure 5d).

The free form of JC-1, a cationic green fluorescence dye, enters and accumulates in negatively charged mitochondria and forms red fluorescent J-aggregates in healthy cells with a normal mitochondrial membrane potential. Compared with the group treated with only H_2_O_2_, the THSG-treated groups showed decreased JC-1 fluorescence (green) but increased J-aggregates (red) (Figure 5e). This result suggests that THSG can prevent the H_2_O_2_-induced loss of the mitochondrial membrane potential, which is indicative of suppression of the mitochondrial pathway of apoptosis.

### 3.6. 2,3,4′,5-Tetrahydroxystilbene-2-O-β-D-Glucoside Enhances Nrf2 Translocation into the Nucleus and Induces mRNA and Protein Expression of Antioxidant/Detoxifying Enzymes under H_2_O_2_-Induced Oxidative Stress Conditions

**Figure 6:**
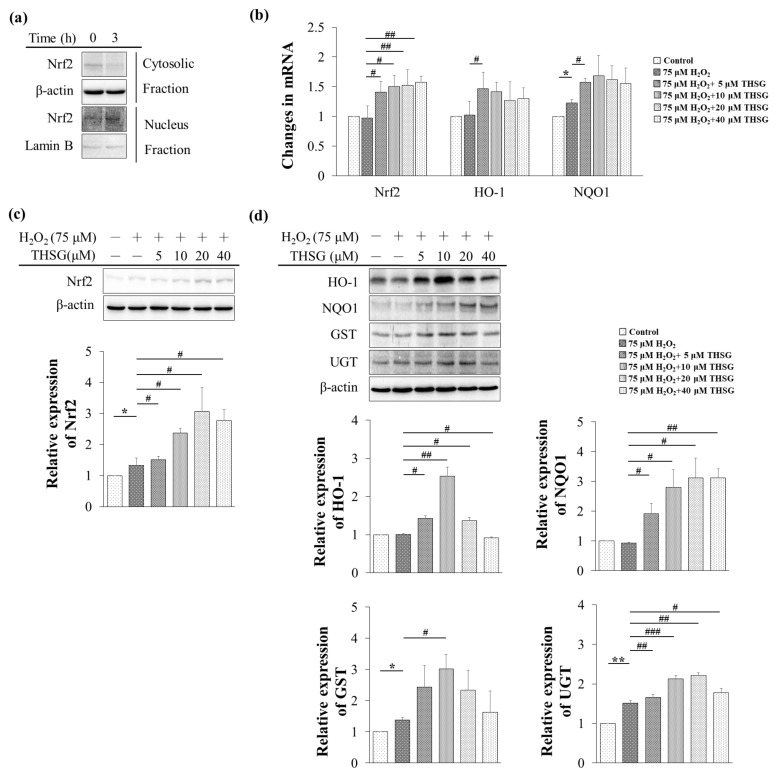
THSG enhances Nrf2 translocation into the nucleus and induces the mRNA and protein expression of antioxidant/detoxifying enzymes under H_2_O_2_-induced oxidative stress conditions. (**a**) The cells were treated with 20 μM THSG for 3 h, and the protein level of Nrf2 in the cytosolic and nuclear fractions was determined by Western blotting. β-actin was used as a cytoplasmic marker and lamin B was used as a nuclear marker. (**b**) The cells were pretreated with 20 or 40 μM THSG for 6 h and subsequently stimulated with THSG combined with 75 μM H_2_O_2_ for 6 h, and the mRNA expression levels of Nrf2, heme oxygenase-1 (HO-1), and NAD(P)H:quinone oxidoreductase-1 (NQO1) were measured by real-time PCR. The results of real-time PCR were normalized to GAPDH. (**c**) The cells were pretreated with various concentrations of THSG (5–40 μM) for 6 h and subsequently stimulated with THSG combined with 75 μM H_2_O_2_ for 24 h, and the cytosolic protein level of Nrf2 was measured by Western blotting. β-actin was used as a cytoplasmic marker. (**d**) The cells were pretreated with various concentrations of THSG (5–40 μM) for 6 h and subsequently with 75 μM H_2_O_2_ for 24 h, and the protein levels of HO-1, NQO1, glutathione-S-transferase (GST), and UDP-glucuronosyltransferase (UGT) were measured by Western blotting. β-actin was used as the loading control. The results of protein expression were normalized to β-actin. Data are presented as the mean ± SD, *n* = 3. *p < 0.05 and **p < 0.01 indicate significant differences from the control group. **^#^***p* < 0.05, **^##^***p* < 0.01, and **^###^***p* < 0.001 indicate significant differences from the H_2_O_2_-treated group.

Under physiological conditions, Nrf2 binds to Kelch-like ECH-associated protein 1 (keap1) and destabilizes in the cytoplasm, whereas under oxidative stress conditions, Nrf2 dissociates from keap1 and translocates to the nucleus to activate the mRNA expression of antioxidant/detoxifying enzymes. Exposure to THSG for 3 h significantly increased the protein level of Nrf2 in the nuclear fraction (Figure 6a). UB/OC-2 cells were treated with various concentrations of THSG (5–40 μM) under H_2_O_2_-induced oxidative stress conditions, and this treatment significantly increased the mRNA expression of HO-1 and NQO1 (Figure 6b). THSG also increased the Nrf2 mRNA and protein levels under H_2_O_2_-induced oxidative stress conditions (Figure 6c,d). As shown in Figure 6d, a protein analysis of THSG-treated UB/OC-2 cells demonstrated significant increases in the levels of antioxidant/detoxifying enzymes (HO-1, NQO1, GST, and UGT) under H_2_O_2_-induced oxidative stress conditions (Figure 6d). Taken together, the results suggest that THSG can increase the capacity of antioxidant defenses against oxidative stress.

## 4. Discussion

Free radicals might affect multiple intracellular processes, including DNA, proteins, cell surface receptors, and membrane lipids. In the cochlea, ROS also induce lipid peroxidation, leading to cell death [3]. Exogenously added H_2_O_2_, which generates a very high level of ROS, might rapidly diffuse across membranes and immediately increase the ROS levels. An extremely high level of ROS directly induces autophagy and apoptosis (Figure 4 and Figure 5).

The THSG, as a chemopreventive agent, protects cells from ROS damage with minimal cytotoxicity [11,14,28]. The free radical scavenging ability of THSG determined using the DPPH assay was similar to that of ascorbic acid (Figure 1). As the chemical structure of THSG is composed of stilbene and glucoside, these compounds might directly scavenge many free radicals. The current study also showed that THSG at concentrations as high as 40 μM did not exert cytotoxic effects in UB/OC-2 cells within 48 h (Figure 2b).

Previous studies have shown that the cytoprotective effects of THSG are mediated by antioxidant/detoxifying enzymes and proteins such as HO-1, NQO1, and GSH. THSG protects against doxorubicin-induced nephropathy and cardiotoxicity by decreasing the ROS levels and inhibiting apoptotic signaling pathways in vivo and in vitro [29,30,31]. The current study also showed that THSG protected against H_2_O_2_-induced cell damage by attenuating oxidative stress in mouse cochlear UB/OC-2 cells. The current study demonstrated that exposure to THSG for 3 h enhanced Nrf2 translocation into the nucleus in pretreated UB/OC-2 cells prior to the addition of H_2_O_2_ (Figure 6a), which resulted in increased Nrf2, HO-1, and NQO1 mRNA and protein expression (Figure 6b–d). The results demonstrated that THSG exhibited antioxidant/detoxifying ability by activating the Nrf2 signaling pathway and increasing HO-1, NQO1, UGT, and GST enzyme expression (Figure 6d). One possible explanation for the biphasic response of HO-1 and GST protein expression could result from the stimulated response of THSG that occurs before 24 h at 20 and 40 μM THSG-treated groups. H_2_O_2_, which increased oxidative stress, directly induced HO-1 expression, and the expression of UGT, GST, and NQO1, which were highly dependent on the Nrf2-ARE pathway, might be induced by THSG through activation of the Nrf2 pathway. Therefore, the findings imply that THSG plays a potential role in protecting against hearing loss through its antioxidant effects.

The THSG attenuated H_2_O_2_-induced autophagy by reducing the LC3-II protein level (Figure 4d). The MDC staining labels autolysosomes and early autophagic compartments, and the accumulation of MDC vesicles corresponds to autophagy [32]. The current study showed that the THSG-treated cells presented lower MDC vesicle accumulation compared with the cells treated with only H_2_O_2_ (Figure 4e). In contrast, THSG reversed the loss of mitochondrial membrane potential, the activation of caspase-3 and PARP-1, and the balance of pro- and anti-apoptotic members of the Bcl-2 family induced by H_2_O_2_ [14]. In agreement with previous studies, the levels of cleaved-caspase 9, cleaved-caspase 3, and cleaved-PARP were increased in H_2_O_2_-stimulated UB/OC-2 cells, but the levels of these apoptosis-related proteins were reduced in THSG-treated UB/OC-2 cells (Figure 5c). The flow cytometry analysis of cell death based on Annexin V/PI staining also showed that THSG protected UB/OC-2 cells against H_2_O_2_-induced apoptosis (Figure 5d). In agreement with previous studies, our results also suggested that THSG prevented cell death by suppressing the mitochondrial apoptotic signaling pathway.

Previous studies found that THSG can restore or reverse mitochondrial biogenesis and mitochondrial function in mouse neuronal cells and RAW 246.7 macrophages through many different anti-inflammatory signaling pathways [30,31]. The JC-1 staining results obtained in our study also showed that THSG might have the ability to stabilize the mitochondrial membrane potential under conditions of H_2_O_2_-induced free radical damage in UB/OC-2 cells (Figure 5e). JC-1 is a cationic dye that enters and accumulates in negatively charged mitochondria and forms a red fluorescent JC-1 dimer that aggregates in healthy cells with a normal membrane potential [33]. Conversely, because an increased mitochondrial membrane permeability results in less negative mitochondria in unhealthy or apoptotic cells, the JC-1 dye does not accumulate to sufficiently high levels to reach the high concentration required for the formation of JC-1 monomer aggregates and continues to exhibit a green fluorescence [33]. H_2_O_2_ rapidly depolarized the mitochondrial membrane potential, which indicated mitochondrial dysfunction. THSG pretreatment relieved this dysfunction by stabilizing the mitochondrial membrane permeability and maintaining the negative charge. Taken together, the results suggest that apoptotic responses might reflect direct changes in mitochondrial permeability caused by ROS formation. The present study demonstrated that THSG suppressed H_2_O_2_-induced UB/OC-2 cell apoptosis by stabilizing the mitochondrial membrane potential and by inhibiting the initiation of the mitochondrial-dependent apoptosis pathway.

## 5. Conclusions

**Figure 7:**
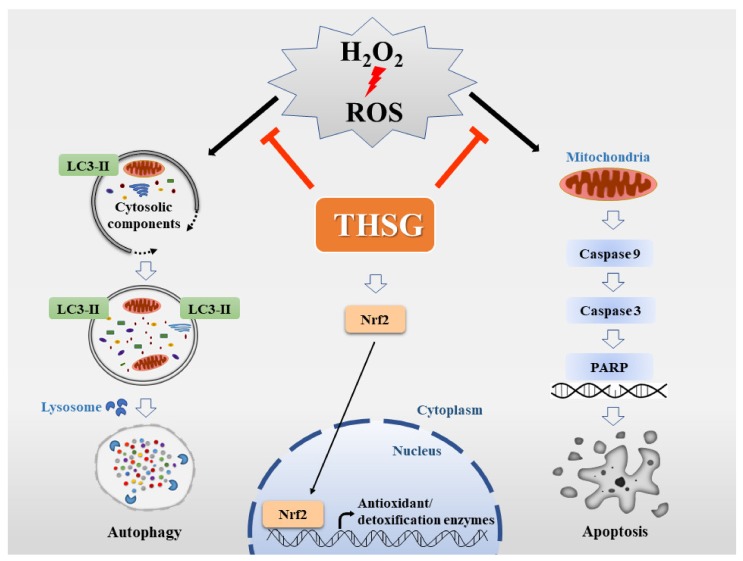
Schematic representation of the possible effect of THSG on the Nrf2 antioxidant pathway and oxidative stress-induced cell death. THSG confers protection against H_2_O_2_-treated mouse cochlear UB/OC-2 cells by upregulating the Nrf2 antioxidant pathway and downregulating autophagy and apoptosis.

In summary, THSG, as a cytoprotective agent, protects against H_2_O_2_-mediated oxidative stress in UB/OC-2 cells by inhibiting both autophagy and the apoptosis pathway. The THSG also directly scavenges free radicals and upregulates antioxidant/detoxifying enzymes to protect against oxidative damage (Figure 7). As THSG is composed of stilbene and glucoside, which contain many polar hydroxyl groups, THSG might activate the Nrf2 signaling pathway through the mitogen-activated protein kinase/extracellular signal-regulated kinase pathway rather than by permeating into cells. The results suggest that THSG might have the ability to protect UB/OC-2 cells from oxidative stress. Since THSG is composed of stilbene and glucoside, which contain many polar hydroxyl groups, THSG may not permeate into cells. Therefore, the detailed molecular mechanisms and signaling transduction pathways underlying both the cytoprotective effects of THSG and its restoration of mitochondrial function should be studied in the future.
